# Crystal structure of caspase-11 CARD provides insights into caspase-11 activation

**DOI:** 10.1038/s41421-020-00201-w

**Published:** 2020-10-13

**Authors:** Muziying Liu, Kang Zhou, Zhihao Xu, Huan Ma, Xiaocong Cao, Xueying Yin, Weihong Zeng, Ayesha Zahid, Sicheng Fu, Kang Ni, Xiaodong Ye, Ying Zhou, Li Bai, Rongbin Zhou, Tengchuan Jin

**Affiliations:** 1 Department of Obstetrics and Gynecology, The First Affiliated Hospital of USTC, Division of Life Sciences and Medicine, University of Science and Technology of China, No.17 Lujiang Rd, Hefei, Anhui 230001 China; 2 Hefei National Laboratory for Physical Sciences at Microscale, CAS Key Laboratory of Innate Immunity and Chronic Disease, School of Basic Medical Sciences, Division of Life Sciences and Medicine, University of Science and Technology of China, Hefei, Anhui 230001 China; 3 Hefei National Laboratory for Physical Sciences at the Microscale, Department of Chemical Physics, University of Science and Technology of China, Hefei, Anhui 230026 China; 4 CAS Center for Excellence in Molecular Cell Science, Shanghai, 200031 China

**Keywords:** Innate immunity, X-ray crystallography

## Abstract

Murine caspase-11 is the centerpiece of the non-canonical inflammasome pathway that can respond to intracellular LPS and induce pyroptosis. Caspase-11 contains two components, an N-terminal caspase recruitment domain (CARD) and a C-terminal catalytic domain. The aggregation of caspase-11 is thought to promote the auto-processing and activation of caspase-11. However, the activation mechanism of caspase-11 remains unclear. In this study, we purified the caspase-11 CARD fused to an MBP tag and found it tetramerizes in solution. Crystallographic analysis reveals an extensive hydrophobic interface formed by the H1–2 helix mediating homotypic CARD interactions. Importantly, mutations of the helix H1–2 hydrophobic residues abolished the tetramerization of MBP-tagged CARD in solution and failed to induce pyroptosis in cells. Our study provides the first evidence of the homotypic interaction mode for an inflammatory caspase by crystal model. This finding demonstrates that the tetramerization of the N-terminal CARD can promote releasing of the catalytic domain auto-inhibition, leading to the caspase-11 activation.

## Introduction

Inflammasome signaling pathways sense different body injuries and pathogen infections, and are essential components of the innate immune system^1^. Unlike several NOD-like receptors (NLRs) and AIM2-like receptors (ALRs) that, in response to certain Pathogen Associated Molecular Pattern (PAMPs), form a canonical inflammasome complex for activation caspase-1, the non-canonical pathway can directly respond to the cytosolic bacterial lipopolysaccharide (LPS), a molecule that plays an important role in endotoxic shock^2–8^.

Murine caspase-11 and its human orthologs caspase-4/5 are essential for the non-canonical inflammasome pathway^9^. Sensing of cytoplasmic LPS results in the self-cleavage and activation of caspase-11^10^. The activated caspase can then drive the canonical inflammasome pathway to induce pyroptosis^9,11–13^. The activation of caspase-11 is involved in the development of inflammatory responses, such as lethal sepsis, making it an important target for drug development^10^.

Caspase-11, -4, and -5 belong to a family of aspartate-specific cysteine proteases. Their activation requires oligomerization and autoproteolysis. Caspase-11, -4, and -5 contain an N-terminal death fold named caspase recruitment domain (CARD) and a C-terminal catalytic domain^14^. The CARD mediates caspase oligomerization through CARD–CARD interaction. Then, proximity-induced autoproteolysis separates the pro-domain from the catalytic domain (p30) and further separates the catalytic domain into a large and a small catalytic unit (p20 and p10), activating the caspase protein^15,16^. The CARD of caspase-11 was found to be essential for LPS-induced pyroptosis^11^. However, the structural mechanism of the caspase-11 CARD–CARD interactions remains unexplored.

Here we describe the crystal structure of caspase-11 CARD. The structure reveals that the caspase-11 CARD undergoes tetramerization mediated by the hydrophobic interface in the H1–2 helix. Further mutagenesis experiments suggest that the hydrophobic interface is critical for the oligomerization of CARD and self-cleavage of caspase-11 in cells.

## Results

### MBP-tagged caspase-11 CARD exists as tetramers in solution

**Fig. 1:**
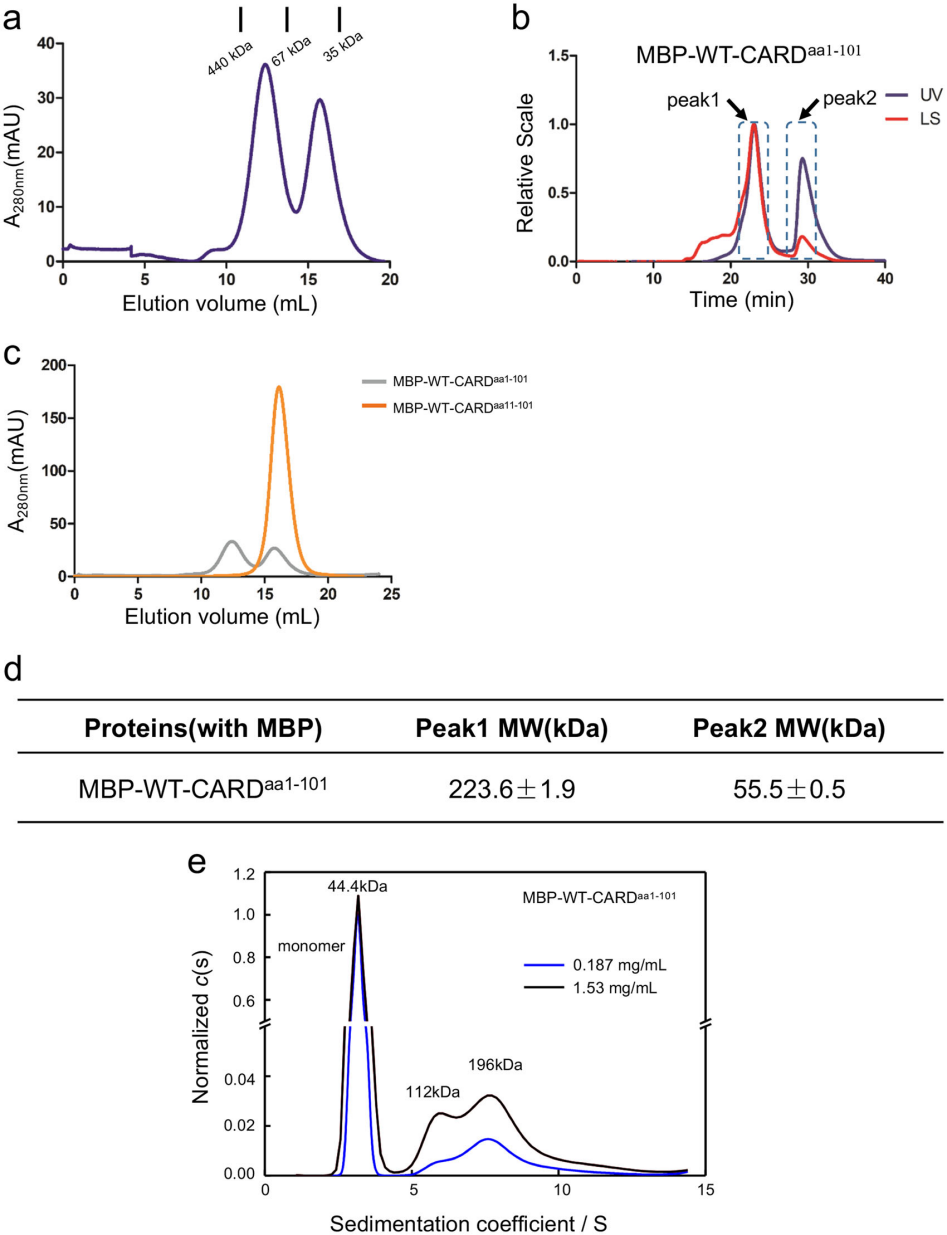
Wild-type caspase-11 CARD is partially tetramerized in solution. **a** *E. coli-*expressed, MBP-tagged WT-CARD^aa1–101^ protein elutes as two peaks, polymer and monomer. **b** Multi-angle light scattering (MALS) analysis of the monomer/oligomer peak. **c** Gel-filtration chromatography of WT-CARD^aa1–101^ and WT-CARD^aa11–101^ proteins (1 mg each). **d** ASTRA analysis of the molecular weights of different peaks in B. **e** Sedimentation velocity AUC profiles and the c(s) distributions for WT-CARD^aa1–101^ (at 0.187, 1.53 mg/mL).

The functional motif of caspase-11 CARD is reported to reside in amino acid residues 1–59 of caspase-11 protein^11^, although the canonical CARD region is predicted to be within residues 1–80. To characterize the biochemical behavior, we generated an expression construct encoding the first 101 amino acids (aa1–101) of caspase-11 with an maltose-binding protein (MBP) tag on the N terminus (WT-CARD^aa1–101^). When purified from *Escherichia coli*, the WT-CARD^aa1–101^ with the MBP tag eluted with two peaks in a size-exclusion chromatogram (SEC). One peak represents polymers, probably resulting from misfolded samples, and the other represents a smaller sized species corresponding to a monomer (Fig. 1a). We further collected the protein samples in each peak to analyze their oligomerization states by static light scattering. The pooled monomer peak sample demonstrated a dynamic re-distribution between two different species (Fig. 1b, c), corresponding to tetramers and monomers (Fig. 1d). The continuous sedimentation coefficient distributions curves, c(s), for WT-CARD^aa1–101^ from monomer peak in SEC was obtained. The molecular weight of each peaks revealed a state switch from monomer to homodimer and homotetramer of WT-CARD^aa1–101^, which also showed the state switch of WT-CARD^aa1–101^ was in a concentration-dependent manner (Fig. 1e). Interestingly, the deletion of the first ten amino acids on the N terminus led to a stable monomer of CARD^aa11–101^ in solution (Fig. 1c).

### Overall structure of the caspase-11 CARD in crystal

**Fig. 2:**
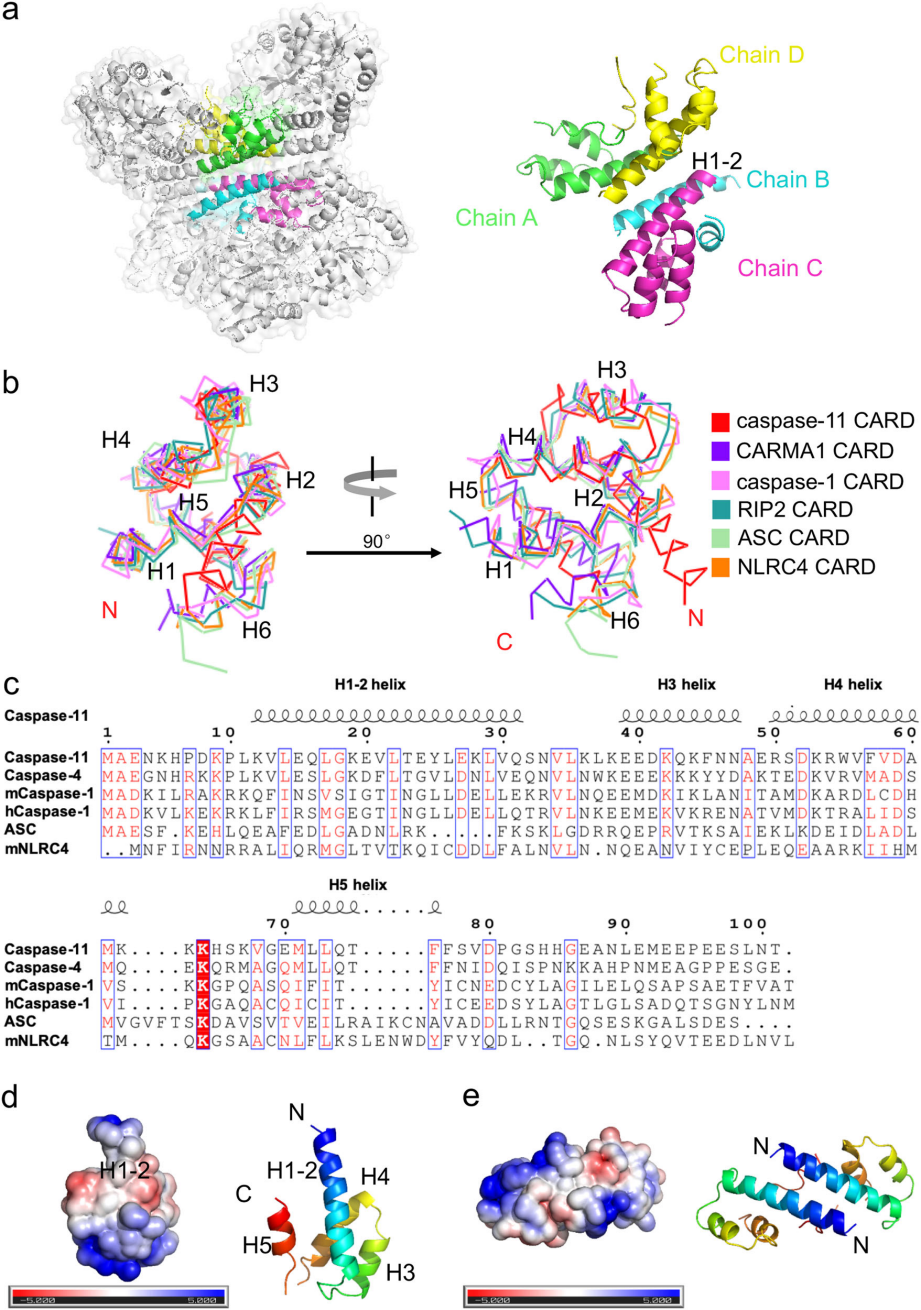
Overall structure of caspase-11 CARD. **a** Homotetrameric packing of caspase-11 CARD in crystal. The H1–2 helices reside in the center of the interaction interfaces. **b** A stereo diagram of superimposed known CARD structures: CARMA1-CARD (PDB ID: 4jup), caspase-1-CARD (PDB ID: 5fna), RIP2-CARD (PDB ID: 6ggs), ASC-CARD (PDB ID: 5gpq), NLRC4-CARD (PDB ID: 6n1i), and caspase-11 CARD. **c** Sequence alignment of residues 1–101 of caspase-11, caspase-4, mcaspsae-1, hcaspase-1, ASC, and mNLRC4. Different amino acids are in white and conserved residues are highlighted in red. The secondary structure of caspase-11 CARD is shown above the sequence. **d** An electrostatic surface of Chain C generated with APBS software. Negatively charged regions are represented in red, neutral regions in white, and positively charged regions in blue. Locations of the hydrophobic interface are indicated. The N terminus sequence of H1–2 contains neutral amino acids (white). The direction of the chain (N–C terminus) is colored as blue to red. **e** An electrostatic surface representation of Chain A and D generated with APBS software. Negatively charged regions are represented in red, neutral regions in white, and positively charged regions in blue. Locations of the hydrophobic interface are indicated. For each chain, the N terminus sequence of H1–2 contains neutral amino acids (white). The direction of the chain (N–C terminus) is colored as blue to red.

The crystal structure of the caspase-11 CARD (residues 11–101) was determined at a resolution of 2.8 Å using X-ray crystallography with an N-terminal MBP as a crystallization tag (Table 1). This technique has been used successfully in the structure determination of several death domains^17,18^. As shown in Fig. 2a, the caspase-11 CARD contains four chains in one asymmetric unit (chain A, B, C, D). The structure of caspase-11 CARD displays a unique feature that is distinct from all known death domain superfamily members (Fig. 2a), it is composed of four amphipathic α-helices instead of the classical six^19^. Comparison with the classic CARD structure reveals that, in the caspase-11 CARD, the classical helix H1 is fused with H2 to form a long helix that we named as H1–2. The three short helices following the H1–2 are well superimposable with the H3, H4, and H5 of classical death folds, but sequence alignment of the CARD domains shows that helices H1 to H5 are not highly conserved even among the homologous proteins (Fig. 2b, c). H6 cannot be observed in our crystal model (Fig. 2b).

**Table 1:** X-ray data collection and refinement parameters for caspase-11 CARD.

No. of reflections (total/unique)	302,171/51,251
Space group	P2_1_2_1_2_1_
Redundancy^a^	5.89 (5.77)
Unit cell parameters	
* a*, *b*, *c* (Å)	77.31, 145.16, 187.72
* α*, *β*, *γ* (°)	90, 90, 90
No. of measured reflections	302,171 (45,074)
No. of unique reflections	51,251 (7804)
Completeness (%)^a^	97.3 (92.9)
* I*/*σ* (*I*)^a^	20.64 (2.03)
R-means^a^	0.069 (0.826)
CC (1/2)^a^	0.999 (0.808)
Refinement	
Resolution (Å)	50–2.8
No. of protein atoms	12,882
R.m.s.d. bond lengths (Å)	0.011
R.m.s.d. bond angles (°)	1.173
*R*_work_ (%)^b^	30.55
*R*_free_ (%)^c^	34.01
Wilson B-factor	84.15
Ramachandran plot (favored/disallowed)	92.3/1.22
PDB code	6KXG

Values in parentheses are for the highest-resolution shell.

^a^*R*_means_ = Σ_*h*_(*n*/*n* − 1)^1/2^ Σ_*i*_ |*I*_*i*_(*h*) − <*I*(*h*)>| / Σ_*h*_Σ_*i*_*I*_*i*_(*h*), where *I*_i_(*h*) and <*I*(*h*)> are the *i*th and the mean measurement, respectively, of the intensity of reflection *h*.

^b^*R*_work_ = Σ_*h*_ ||*F*_obs_ (*h*)| − |*F*_calc_ (*h*)|| / Σ_*h*_ | *F*_obs_ (_*h*_)|, where *F*_obs_ (*h*) and *F*_calc_ (*h*) are the observed and calculated structure factors, respectively. No *I*/*σ* cutoff was applied.

^c^*R*_free_ is the *R*-value obtained from a test set consisting of a randomly selected 5% subset of the data. The test set is excluded from refinement.

The electrostatic surface analysis of the CARD with acidic, basic, and hydrophobic patches indicates that several neutral amino acids in the long H1–2 (residues L372–S391) form a hydrophobic surface, which possibly mediates the oligomerization of CARD (Fig. 2d). Moreover, the interactions between the four protomers are primarily mediated by H1–2 (Fig. 2e). PISA (Proteins, Interfaces, Structures, Assemblies) server^20^ was used to analysis the molecular interface in the structure. We found that the interface area between chain B and chain C is 1058.2 Å^2^, whereas the area is 993.0 Å^2^ between chain A and chain D, with a negative Δ^i^G indicating a strong hydrophobic interaction between two chains. Although the interface area reduced sharply between chain A and chain B, and between chain C and chain D, the area is even less between chain A and chain C, and between chain B and chain D, suggesting that B/C and A/D formed homodimers before aggregating into a tetramer (Table 2). The presence of homodimer and homotetramer are also revealed by sedimentation velocity analytical ultracentrifugation (SV-AUC) analysis of WT-CARD^aa1–101^ (Fig. 1e). All of these intermolecular interactions are primarily mediated by the H1–2 helix. Collectively, these data suggest the H1–2 of the caspase-11 CARD reveals by crystal structure might be involved in the self-interaction of caspase-11 CARD.

**Table 2:** PISA analysis of the four chains in the crystal structure.

Structure1 : Structure2	Interface area (Å^2^)	Δ^i^G (kcal/mol)	Δ^i^G *P*-value
Chain C : Chain B	1058.2	–18.7	0.361
Chain A : Chain D	993.0	–20.2	0.226
Chain A : Chain B	241.0	–5.8	0.296
Chain C : Chain D	224.9	–4.8	0.210
Chain D : Chain B	83.1	–2.7	0.143
Chain A : Chain C	82.1	–2.6	0.093

Δ^i^G indicates the solvation free energy gain upon formation of the interface, in kcal/mol. Negative Δ^i^G indicates hydrophobic interfaces or positive protein affinity. This value does not include the effect of the satisfied hydrogen bonds and salt bridges across the interface.

*P* > 0.5 means that the interface is less hydrophobic than it should be, therefore the interface is likely to be an artifact of crystal packing. *P* < 0.5 indicates the interface has a higher hydrophobicity than the predicted average for a given structure, implying that the interface surface may be interaction-specific. The limiting case of *P* = 0 means that no other interface of the observed area may have a lower Δ^i^G; therefore, this interface is a truly unique spot on the protein surface.

### Mutations in the hydrophobic interface abolish the aggregation of caspase-11 in vitro

**Fig. 3:**
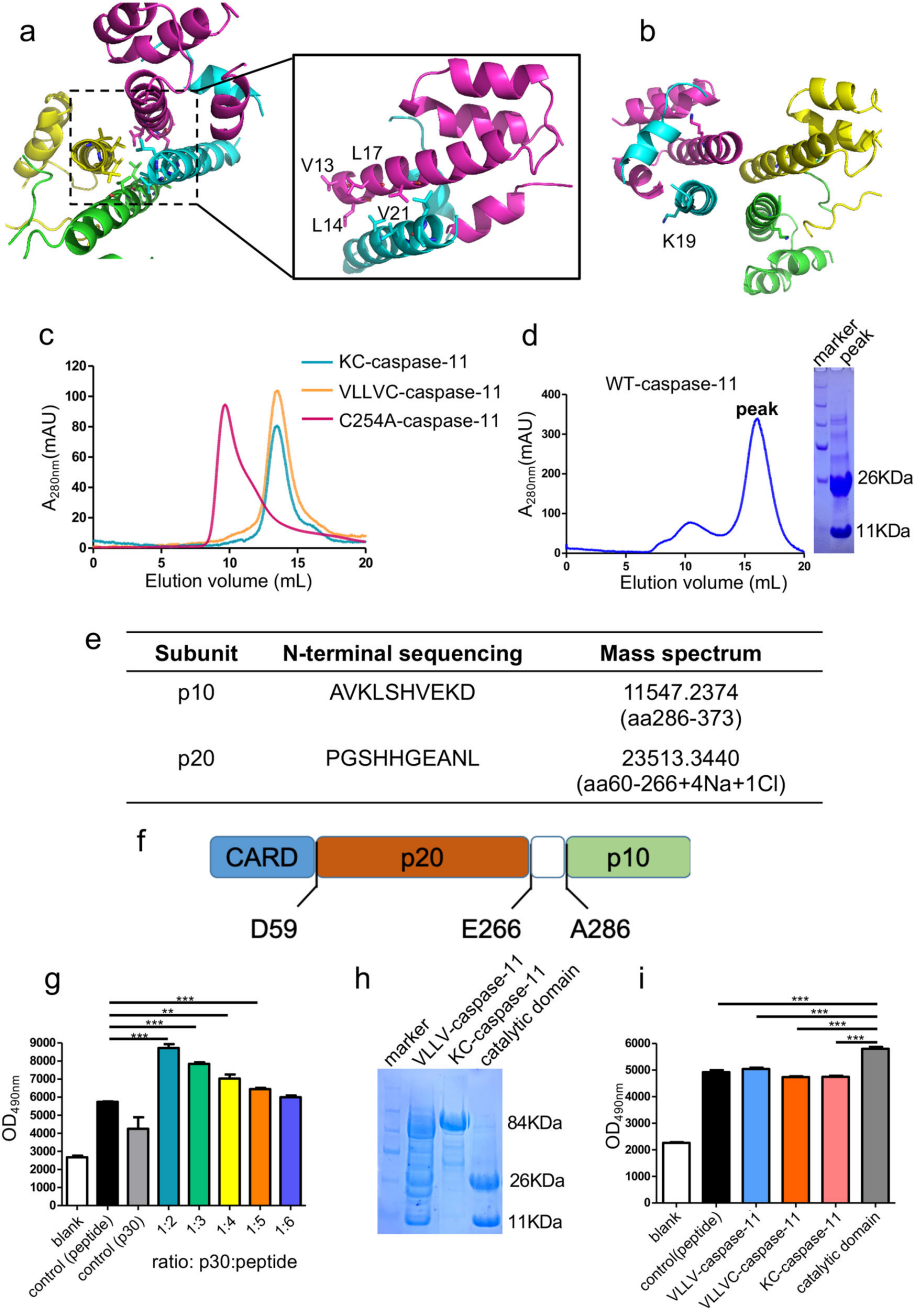
Mutations abolish the aggregation of caspase-11 CARD and prohibit the self-cleavage of caspase-11. **a** The four hydrophobic amino acids on H1–2, V13, L14, L17, and V21, are indicated as sticks. **b** The basic amino acid K19 resides in the middle of H1–2 and is indicated as a stick. **c** Gel-filtration chromatography of *E. coli*-expressed C254A-caspase-11, K19E-caspase-11, and VLLV-caspase-11 (both in K19E-caspase-11 and VLLV-caspase-11 are on the C254A background). **d** *E. coli*-expressed wild-type mature caspase-11 protein (left panel), the corresponding SDS-PAGE gel is shown on the right. **e** Initial ten amino acids detected by N-terminal sequencing of the two bands in **d** transferred to a PVDF membrane and mass spectrum detecting the molecular weight of the two bands in **d**. **f** Diagram of caspase-11 and the self-cleavage sites. **g** Proteolytic activity assay on the predicted caspase-11 catalytic domain. GSDMD cleavage reporter was mixed with different amounts of the predicted caspase-11 catalytic domain. The initial concentration of predicted caspase-11-catalytic domain is 1 μM. All data shown are representative of at least three independent repeats. **h** VLLV mutation reduces self-cleavage of full-length caspase-11 revealed by SDS-PAGE analysis. **i** Synthetic DABYL-GQLSLLSDGID-glu (edans) (GSDMD cleavage reporter) was used as the substrate of mature caspase-11 and the concentration ratio of caspase-11 proteins to the reporter is 1 : 5. All data shown are representative of at least three independent repeats.

To investigate whether the activation of caspase-11 relies on the homotypic interaction of CARD as observed in the crystal structure, we generated four mutations, V13A, L14A, L17A, V21A (VLLV) on the H1–2. These residues are all located in the N-terminal side of CARD domain in order to destroy the hydrophobic interaction (Fig. 3a). Compared with the charge mutation K19E, which positions in the middle of helix H1–2 (Fig. 3b) as mentioned in a previous study^11^, VLLV mutations on the caspase-11 CARD only change the side chains of the hydrophobic amino acids, but not their charges. C254A-caspase-11 containing the VLLV or K19E mutations in their H1–2s with the MBP tag, when purified from *E. coli*, were both eluted as monomers from the gel-filtration columns, whereas the MBP-caspase-11 protein carrying only the C254A mutation retained its ability to form oligomer with multiple polymerization states (Fig. 3c). This result demonstrates that the hydrophobic region of the CARD is required for the oligomerization of caspase-11.

The abolished oligomerization ability in H1–2 mutant caspase-11 proteins prompted us to hypothesize that these mutations may also affect caspase-11 activation. A previous report showed that the wild-type caspase-11 protein failed to express in *E. coli* system^13^. Considering this, we used an MBP tag to stabilize caspase-11 in *E. coli* to investigate the caspase-11 activation. A ~37 kDa fraction was eluted from an analytical gel-filtration column and the fraction was further separated into two bands on an SDS gel (Fig. 3d). With N-terminal sequencing and mass spectrum, we confirmed that the bands on the SDS gel were the p10 and p20 subunits of the caspase-11 catalytic domain (Fig. 3e). The natural cleavage site of CARD is between the 59th and the 60th residue of caspase-11, and the two mature subunits of the catalytic domain (p30), p20 and p10, span residues 60–266, and 286–373, respectively (Fig. 3f). Activated caspase-11 cleaves gasdermin D (GSDMD) that can lead to pyroptosis and interleukin-1β maturation^11,12^. Therefore, to directly test the proteolytic activities in the wild-type and VLLV mutant caspase-11, we synthesized a self-quenched fluorescent peptide DABYL-GQLSLLSDGID-glu(edans) that mimics the GSDMD cleavage site for caspase-11. This peptide can be used as a reporter for caspase-11 proteolytic activity (GSDMD cleavage reporter). We incubated auto-proteolytic wild-type p30 protein fraction with the GSDMD cleavage reporter. With the increased ratio of p30 to the GSDMD cleavage reporter, the fluorescent product increased, indicating that the fraction is proteolytically active (Fig. 3g). Then, we introduced the VLLV mutations to the wild-type caspase-11 and purified the protein from *E. coli* (VLLV-caspase-11). SDS-polyacrylamide gel electrophoresis analysis showed that the VLLV mutation reduced the self-cleavage of full-length caspase-11 (Fig. 3h). Furthermore, we found that the proteolytic activity of VLLV-caspase-11 was greatly reduced (Fig. 3i). These results show that the VLLV mutation on CARD not only affect the aggregation of caspase-11 but also inhibit the cleavage of caspase-11 itself and substrates.

Together, these data indicate that the hydrophobic interface on the CARD helix H1–2 region is essential for the aggregation of caspase-11 that leads to caspase maturation and downstream signal cascade activation.

### Caspase-11-mediated GSDMD cleavage and cell death are dependent on the hydrophobic surface on H1–2 of CARD

**Fig. 4:**
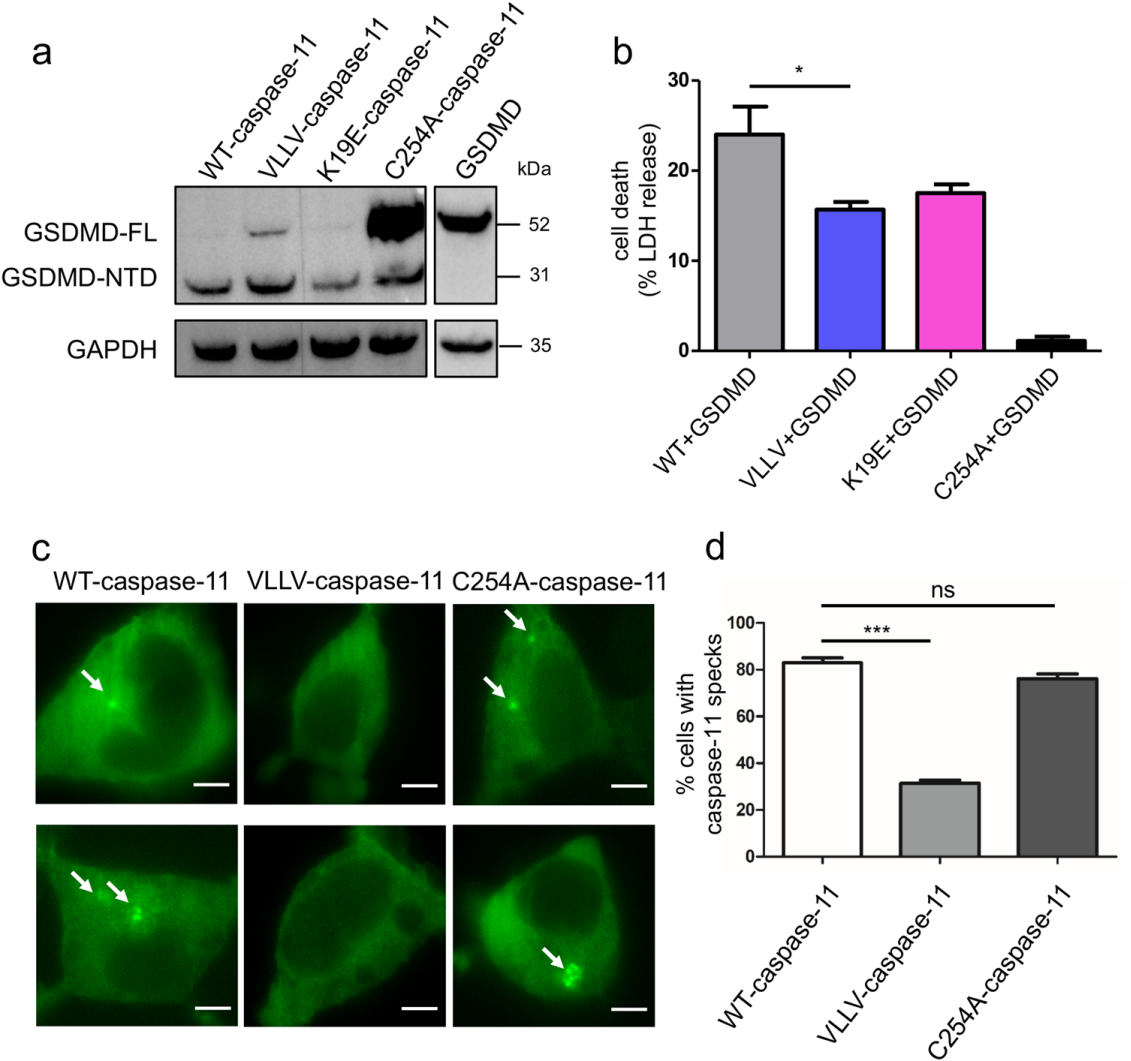
The hydrophobic interface is critical for the cellular activation of the caspase-11 inflammasome. **a** Immunoblot analysis of GSDMD and GAPDH in the HEK293T cell lysates after 24 h of transfection with caspase-11 and GSDMD. **b** Same experiment as in **a**, cell death was monitored by LDH release in HEK293T cell. Data are shown as mean ± SEM (*P < 0.05, two-tailed *t*-test). **c** Caspase-11-EGFP speck formation (arrows) in HEK293T cells at 24 h post transfection (scale bars = 5 μm). **d** Quantitative comparison of caspase-11-EGFP speck formation in **c**. Quantified values are shown as mean ± SEM (≥50 cells per group were pooled from three independent experiments. *P < 0.05, **P < 0.01, and ***P < 0.001, two-tailed *t*-test).

As we found that purified VLLV-caspase-11 fails to activate, we further sought to evaluate the activity of wild-type and mutant caspase-11 in cells. As caspase-4 is not expressed in HEK293T cells (Supplementary Fig. S1), we co-transfected hGSDMD with full-length caspase-11 plasmids, i.e., WT-caspase-11, VLLV-caspase-11, K19E-caspase-11, or C254A-caspase-11, into HEK293T cells. Without any stimulation, WT-caspase-11 underwent self-activation, then the hGSDMD was cleaved into two fragments (Fig. 4a). In addition, cell death was significantly upregulated (Fig. 4b). In summary, compared to WT-caspase-11, C254A-caspase-11 displayed a drastic decrease in the hGSDMD cleavage and cell death, and the VLLV-caspase-11 mutant displayed a partial decrease in both GSDMD cleavage and cell death.

We further tested whether the overexpressed wild-type and mutant caspase-11 could nucleate oligomeric assemblies of the caspase-11 protein. For this, we constructed wild-type and mutant caspase-11 expression plasmids tethered with enhanced green fluorescent protein (EGFP) on the C terminus (WT-caspase-11-EGFP, VLLV-caspase-11-EGFP, and C254A-caspase-11-EGFP). When transfecting 293T cells with WT-caspase-11-EGFP or C254A-caspase-11-EGFP, we observed caspase-11-EGFP specks that indicate caspase-11 aggregations. In comparison, significantly less percentage of cells transfected with the VLLV-caspase-11-EGFP had caspase-11-EGFP specks (Fig. 4c, d). These results demonstrate that the caspase-11-CARD mutations can affect the oligomerization and the activation of caspase-11 in cells.

### LPS does not associate with the MBP-tagged caspase-11 CARD domain in solution

**Fig. 5:**
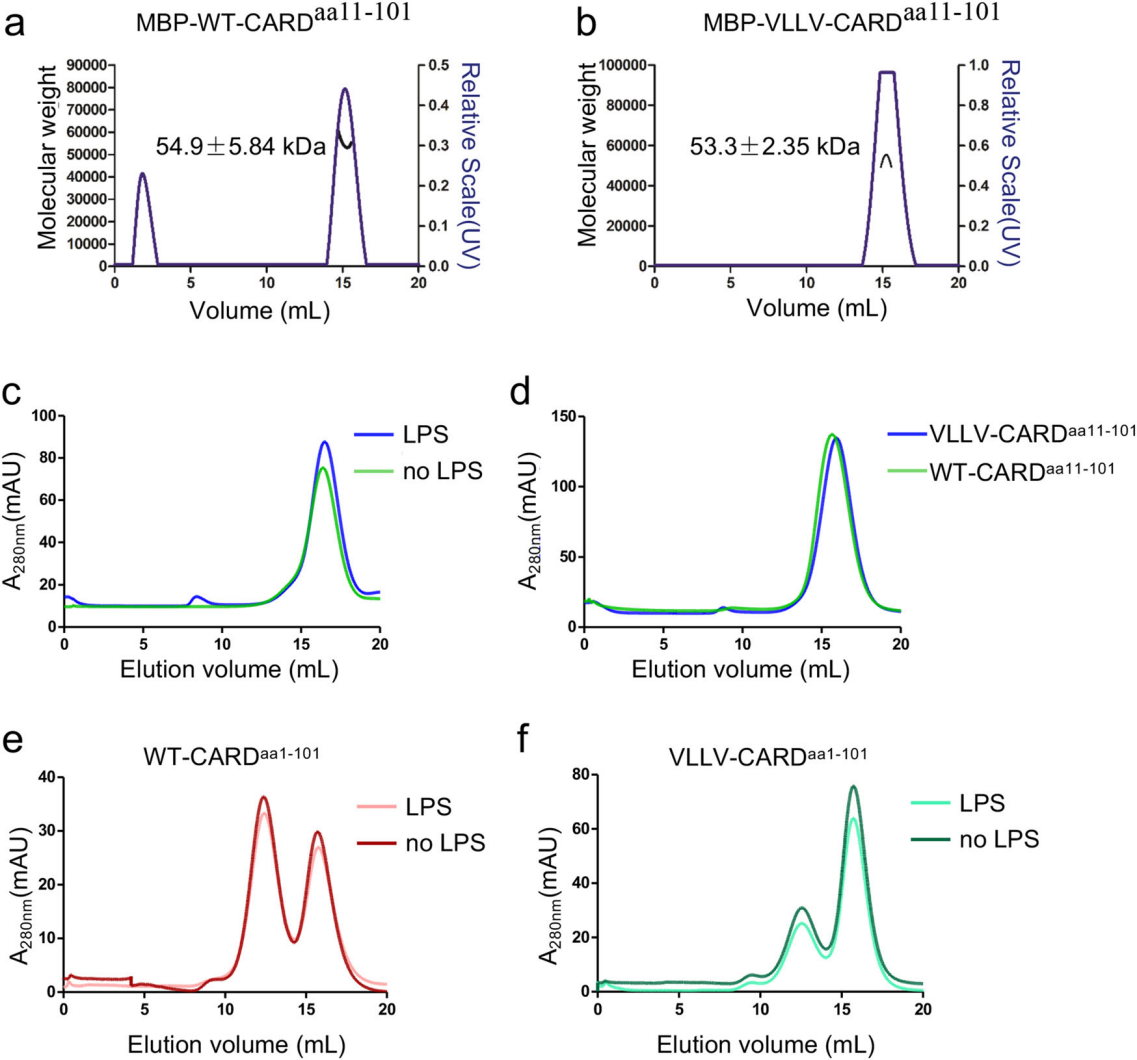
LPS fails to induce the aggregation of wild-type and mutant caspase-11 CARDs. **a**, **b** Multi-angle light scattering (MALS) profile. The black line indicates the experimentally calculated molecular weight. **c** Purified caspase-11 WT-CARD^aa11–101^ protein with a TEV recognition sequence between MBP fusion protein and CARD. Protein (1 mg/mL) was incubated with 5 μg LPS at 4 °C for 4 h. **d** Purified WT-CARD^aa11–101^ and VLLV-CARD^aa11–101^ with MBP tag (1 mg each) incubated with 5 μg LPS at 4 °C for 4 h. **e**, **f** Purified WT-CARD^aa1–101^ and VLLV-CARD^aa1–101^ with MBP tag (1 mg each) incubated with or without 5 μg LPS at 4 °C for 4 h.

The above results showed that the homotypic CARD interaction interface is critical for caspase-11 activation and overexpressing caspase-11 proteins in HEK293T cells could directly activate the non-canonical inflammasome signaling pathway without LPS stimulation through CARD-mediated oligomerization of caspase-11. Previous research suggests that the LPS binds to the CARD domain of caspase-4, which promotes caspase-4 activation^11^, but another research indicates that LPS binding requires both CARD and catalytic domain of caspase-11^21^. We next tested whether LPS is required for the aggregation of caspase-11 CARD in solution. The deletion of residues 1–59 of caspase-11 was shown to compromise the LPS-induced caspase-11 activation^11^. Here we used the MBP fusion protein to stabilize the caspase-11 CARD and the mutant VLLV-CARD in the *E. coli* expression system, with a ~10 residue linker (TEV cleavage sequence) between MBP and caspase-11 CARD proteins. Caspase-11 CARD^aa11–101^ and VLLV-CARD^aa11–101^ formed monomers during purification, which was confirmed by SEC-multi-angle light scattering (MALS) (size-exclusion chromatography followed by MALS (Fig. 5a, b). After incubation with LPS at 4 °C for 4 h, the gel-filtration profile displayed no change (Fig. 5c). Compared with VLLV-CARD^aa11–101^, both proteins failed to respond to LPS stimulation (Fig. 5d). Similar to WT-CARD^aa11–101^ and VLLV-CARD^aa11–101^, LPS failed to induce the polymerization of WT-CARD^aa1–101^ and VLLV-CARD^aa1–101^ (Fig. 5e, f). These data led to the conclusion that LPS does not directly bind with MBP-tagged caspase-11 CARD, despite that it is able to form functional related homo-oligomers.

### Truncations of caspase-11 CARD result in different dynamic state and which is in a linker sequence independent manner

**Fig. 6:**
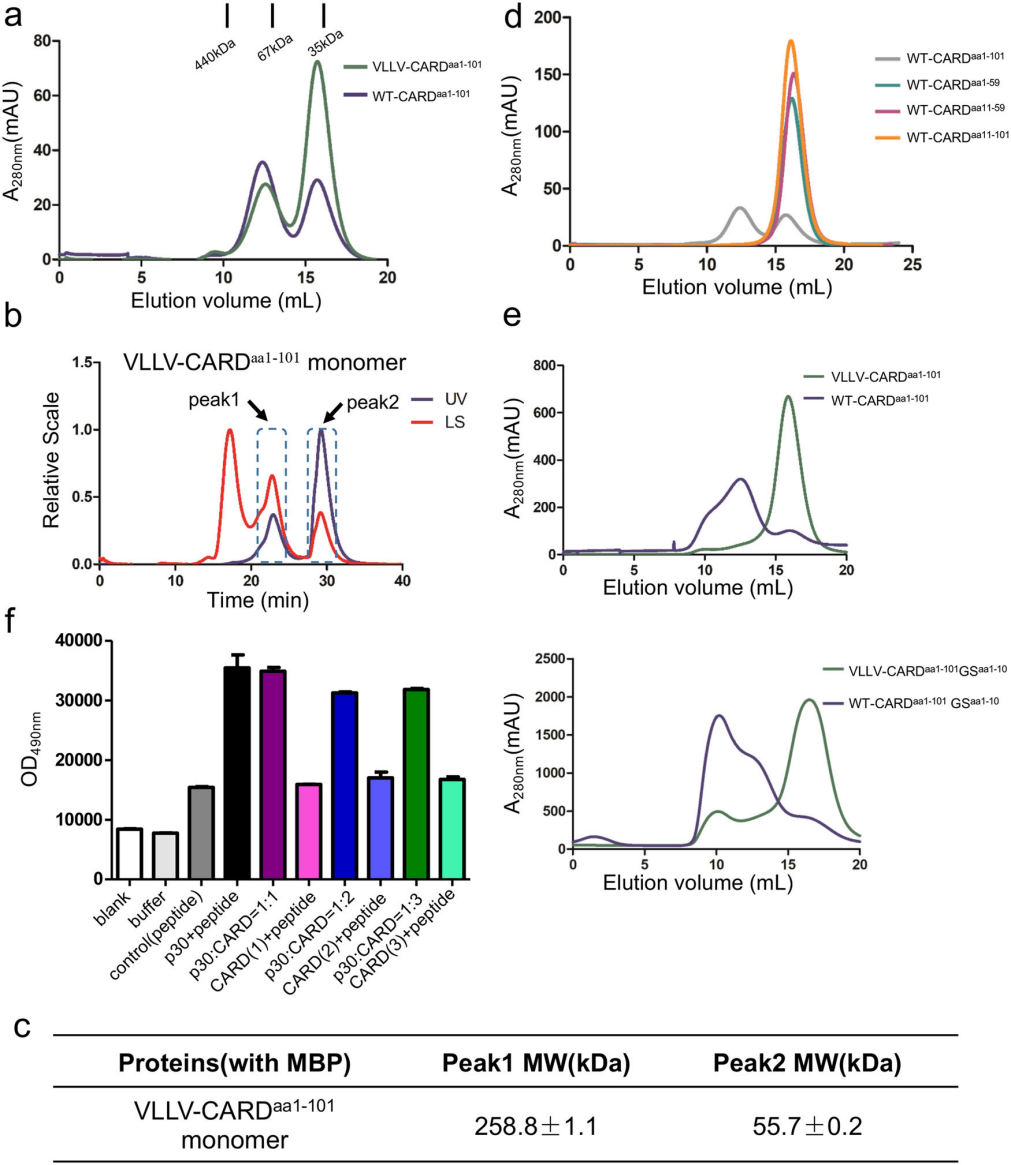
Tetramerization of caspase-11 CARD^aa1–101^ in solution. **a** *E. coli-*expressed MBP-tagged WT-CARD^aa1–101^ and VLLV-CARD^aa1–101^ protein both elute as two peaks. **b** Multi-angle light scattering (MALS) analysis of VLLV-CARD^aa1–101^ monomer peak in **a**. **c** ASTRA analysis of the molecular weight of different peaks in **b**. **d** Gel-filtration chromatography of truncated CARDs with the same quantity of proteins (1 mg). **e** Gel-filtration chromatography analysis of MBP-tagged WT-CARD^aa1–101^ and VLLV-CARD^aa1–101^ with a GS repeat sequence. **f** CARD inhibition of p30 proteolytic activity was detected by the fluorescence derived from the GSDMD cleavage reporter. CARD(1) indicates the concentration ratio between the catalytic domain and CARD protein as 1 : 1; CARD(2) indicates 1 : 2; CARD(3) indicates 1 : 3.

For crystallization, we used N-terminal 10 amino acids deficient CARD (11–101 residues), but it stays as monomer in solution during purification (Fig. 1d). To find out whether CARD aggregation requires all of the residues in the CARD domain, we made several truncations of WT-CARD, WT-CARD^aa1–59^, and WT-CARD^aa11–59^ due to the natural cleavage site of caspase-11 (Fig. 3e). Unfortunately, we failed to obtain the crystal structure of different truncations of CARD. Therefore, we decided to determine the mechanism of oligomerization of CARD based on their structural characteristics. WT-CARD^aa1–101^ showed a tetrameric state in solution during purification (Fig. 1a, b, d). Further, we also detected VLLV-CARD^aa1–101^, similar to WT-CARD^aa1–101^, demonstrates an equilibrium between oligomers and monomers during gel-filtration purification (Fig. 6a, b), even though unlike WT-CARD^aa1–101^, the monomeric state appears to dominate for VLLV-CARD^aa1–101^. Among different truncations, only WT-CARD^aa1–101^ displayed a dynamic equilibrium between tetramers and monomers, whereas other truncations are monomers (Fig. 6c). Therefore, we hypothesized that the first ten residues are critical for its self-association when fused at the C terminus of MBP. To test whether the sequence of the first ten amino acids is required for CARD self-assembly, we replaced the first ten amino acids to a flexible GS repeat sequence (5′-GSGSGSGSGS-3′) in WT-CARD^aa1–101^. Compared with WT-CARD^aa1–101^, the GS repeat mutant showed no change in the tetramer peak (Fig. 6e). This suggests that a flexible loop at the N terminus, regardless of the sequence, is required for the oligomerization of CARD.

We also sought to find out the resting mechanism of caspase-11. We further hypothesized that there might exist some self-interaction between CARD and catalytic domain of caspase-11. Unfortunately, we failed to observe the interaction of the two units of caspase-11 (Fig. 6f). This result indicated there might be some more subtle mechanism of self-inhibition for caspase-11.

## Discussion

**Fig. 7:**
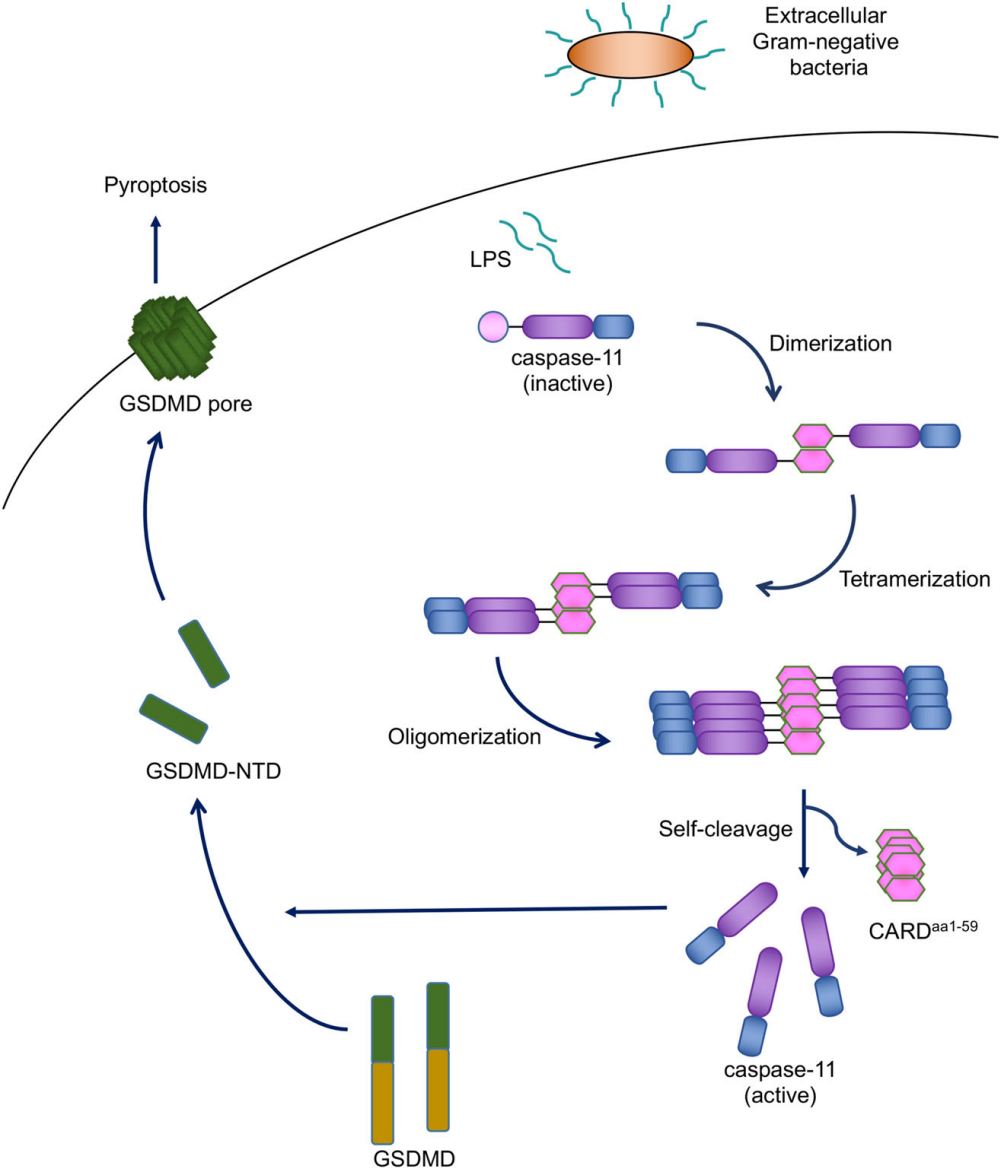
Model of caspase-11 activation. LPS, a component of gram-negative bacteria, can be delivered into the cytosol of host cells. The presence of intracellular LPS results in the activation of the non-canonical inflammasome signaling pathway. The activation of caspase-11 requires the aggregation of CARD from a monomer, via a homodimer, to a homotetramer, then the oligomerization of caspase-11 induces self-cleavage, which results in the release of the catalytic domain of caspase-11. The catalytic domain of caspase-11 then cleaves GSDMD, inducing pyroptosis.

We determined, for the first time, the crystal structure of the CARD domain in an inflammatory caspase. Our data show that the structure of caspase-11 CARD forms an atypical conformation different from other death domain superfamily members. And this conformation indicates a possible activation model of caspase-11 by the hydrophobic interaction mediated by CARD H1–2 (Fig. 7).

Death fold is generally packed loosely^19^ and several death domain members have non-classic structures during activation. For example, DR3 DD H3 and H4 undergo a conformation switch when interacting with tumor necrosis factor receptor-associated death domain^17^; FAD binding can induce the shift of helix H6 to H5 in Fas-associated protein with death domain, which forms a stem helix for the adaptor binding^22^. Therefore, the conformational changes of death domains are often associated with biological functions.

The constructs of caspase-11 CARD and full-length protein both with all mutants are linked with MBP on N-terminal side and His-tag on C-terminal side. The linker (VD) between MBP and the target protein was reported to have minimal impact on the structural and functional characteristics of the target protein^23^. Also, the linker brings the MBP tag closer to the CARD, which can stabilize the protein in the crystal (Supplementary Fig. S2). If removing the MBP tag after purification, we failed to obtain WT-CARD^aa11–101^ (data not shown). In contrast, the mutant VLLV-CARD^aa11–101^ is more stable in solution: it can be purified when the MBP tag in the N terminus is removed using TEV protease (Supplementary Fig. S3).

Inflammatory caspases (caspase-1/4/5/11) all contain a CARD and a catalytic domain^14,24^. Oligomerization caused by CARD–CARD interactions leads to self-cleavage, activation of the catalytic domain, cleavage of GSDMD, and the induction of pyroptosis. Our data show that the homotypic CARD–CARD interface mediated by hydrophobic residues in the elongated H1–2 is critical for the oligomerization of caspase-11 and its activation. Mutations on H1–2 could inhibit the aggregation of caspase-11 and reduce cell death induced by caspase-11. In addition, our results showed that LPS failed to bind with the MBP-tagged caspase-11 CARD. Maybe there also exist an intermediate molecular interaction that directly bind with caspase-11 in response to intracellular LPS stimulation or LPS only educated caspase-11 and then lead to activation. The N-terminal 1–59 residues of caspase-11 were reported to be critical for the activation of caspase-11 induced by LPS^11^. Our study further pinpointed that the H1–2 helix is essential for the oligomerization of caspase-11 during activation. This study of caspase-11 CARD structure and activation mechanism may provide insights into understanding the mechanism of inflammatory caspases in general and future drug development that aims to inhibit inflammasome activation.

Due to technical restrictions, we failed to solve the structure of full-length caspase-11 in resting state. In addition, the exact oligomerization state of full-length caspase-11 remains unknown. It is worth investigating whether the tetramer state observed in our CARD structure is maintained in full-length protein setting.

## Materials and methods

### Plasmids and transfection

Complementary DNA (cDNA) of murine caspase-11 was amplified from the plasmids provided by Feng Shao. The mutant and wild-type caspase-11 cDNAs were inserted into a pEGFP-N1 vector for transient expression in HEK293T cells. A modified pCDNA3.1 vector was used to co-express GSDMD with mutant and wild-type caspase-11 in HEK293T cells. All plasmids were verified by DNA sequencing.

### Purification of recombinant proteins

*E. coli* Rosetta and BL21 (DE3) cells harboring the V28E-CARD plasmid (MBP vector with an N-terminal MBP tag and a C-terminal His6-tag) were grown in Luria-Bertani Broth (LB broth) medium supplemented with 50 μg/mL kanamycin. Protein expression was induced for 4 h at 18 °C with 0.3 mM isopropyl-β-d-thiogalactopyranoside after OD_600_ reached 1.2. The cells were collected by centrifugation and lysed by ultrasonication in a lysis buffer containing 150 mM NaCl, 20 mM imidazole, and 20 mM Tris-HCl (pH 8.0). The cell lysate was clarified by centrifugation at 15,000 × *g* at 4 °C for 20 min and residual debris was removed using a 0.8 μm filter. The supernatant containing the target protein was loaded onto a nickel column (GE Healthcare Life Sciences). The recombinant protein was eluted using the lysis buffer supplemented with 400 mM imidazole. The His6-MBP-tagged protein was further purified through Superdex 200 gel-filtration chromatography (GE Healthcare Life Sciences) in a buffer containing 250 mM NaCl and 10 mM Tris-HCl (pH 8.0).

### Crystallization, X-ray diffraction, structure determination, and refinement

Purified MBP-tagged wild-type CARD protein was concentrated using Amicon centrifugal concentrators (Millipore) to 85 mg/mL. Then, hanging drops were set up for vapor diffusion crystallization. The protein was crystallized at 291 K for a week with a well solution containing 2.4 M ammonium sulfate and 5% isopropanol. Ethylene glycol was added to the reservoir solution to 20% (v/v) as the cryoprotectant and the crystals were flash-cooled in liquid nitrogen for X-ray diffraction data collection.

X-ray diffraction data were collected at the Shanghai Synchrotron Radiation Facility beamline BL17U1^25^. Data were processed with the HKL2000 program suite^26^ and XDS^27^. The structure was determined using molecular replacement method and refined with the Phenix program suite^28^. The MBP structure from the Protein Data Bank (PDB ID: 4IRL) was used as the search model^29^. Model building was performed using Coot (Crystallographic Object-Oriented Toolkit). The structure was validated using the Molprobity server and the RCSB ADIT validation server before deposition. Figures were produced using the program Pymol (Schrödinger).

### Antibodies and reagents

GSDMD antibody was obtained from Sigma-Aldrich (G7422). Glyceraldehyde 3-phosphate dehydrogenase antibody was obtained from ABclona (AC002). Horseradish peroxidase (HRP)-Goat anti-rabbit IgG (D110058) and HRP-Goat anti-mouse IgG (D110087) were purchased from BBI. Unlabeled ultrapure LPS from *E. coli* O111:B4 was purchased from Invitrogen. LDH release kit (C0017) was obtained from Beyotime.

Cell culture products were obtained from HyClone and fetal bovine serum (FBS) was obtained from Gibco.

### Reverse transcription-quantitative PCR

Quantification of caspase-4 expression in 293T cells was performed with SYBR Green I PCR Master Mix (Takara) in the StepOne Plus Real-Time PCR System (ABI). The primers used for each gene examined are listed below. *CASP4*: 5′-CAGACTCTATGCAAGAGAAGCAACGTATGGCAGGA-3′ (forward) and 5′-CACCTCTGCAGGCCTGGACAATGATGAC-3′ (reverse); actin: 5′-CATGTACGTTGCTATCCAGGC-3′ (forward) and 5′-CTCCTTAATGTCACGCACGAT-3′ (reverse).

### In vitro caspase-11 protease activity assay

The p20 and p10 fragments of the wild-type caspase-11 were obtained through expression and the auto-processing of the full-length caspase-11 in *E. coli*. Synthetic self-quenched fluorescence peptide substrate DABYL-GQLSLLSDGID-glu (2 μM, edans), which contains the caspase-11 cleavage site of GSDMD, was incubated with different concentrations of active caspase-11 in 200 μL buffer containing 150 mM NaCl, 1 mM EDTA, 10 mM dithiothreitol, and 50 mM HEPES (pH 7.4). The reactions were incubated for 30 min at room temperature prior to the measurement of fluorescence intensity using a BIOTEK microplate reader (Synergy H1).

### Multi-angle light scattering

MALS measurements were carried out on a DAWN HELEOS 8+ instrument (Wyatt Technology) at room temperature. Caspase-11-CARD protein samples were diluted to 1 mg/mL in a buffer containing 250 mM NaCl and 10 mM Tris-HCl (pH 8.0) for analysis. The data were analyzed with the ASTRA software (Wyatt Technology).

### The sedimentation velocity analytical ultracentrifugation

SV experiments were performed at 50,000 r.p.m. in a Proteomelab XL-A analytical ultracentrifuge (Beckman Coulter Instruments) at 20.0 °C at a wave length of 280 nm (lower concentration) and 250 nm (high concentration) using 12 mm double-sector cells. Protein solution of different concentration were used in a buffer containing 250 mM NaCl and 10 mM Tris-HCl (pH 8.0).

### Total internal reflection fluorescence microscopy

HEK293T cells were transfected with plasmids encoding wild-type or mutant caspase-11. All cells were grown in growth media (high glucose Dulbecco’s modified Eagle’s medium with 10% FBS) and allowed to adhere overnight before transfection. Cells were analyzed 24 h after transfection. All samples were fixed with 4% paraformaldehyde in 1× phosphate-buffered saline for 15 min on ice. Samples were visualized with a Leica SR GSD microscope with a ×160 oil objective and a 100 nm penetration depth.

### Statistical analysis

Data were analyzed using Prism (GraphPad Software, version 5.01).

## Supplementary information

supplemental information, Figures

## Supplementary information

**Supplementary Information** accompanies the paper at (10.1038/s41421-020-00201-w).
