# Supplementary material for: Crystal structure of caspase-11 CARD provides insights into caspase-11 activation
Source: Cell Discov. 2020 Oct 13;6:70. doi: 10.1038/s41421-020-00201-w (PMC7552397; doi:10.1038/s41421-020-00201-w)
Supplement: Supplementary file 1 — supplemental information, Figures [file 41421_2020_201_MOESM1_ESM.pdf]

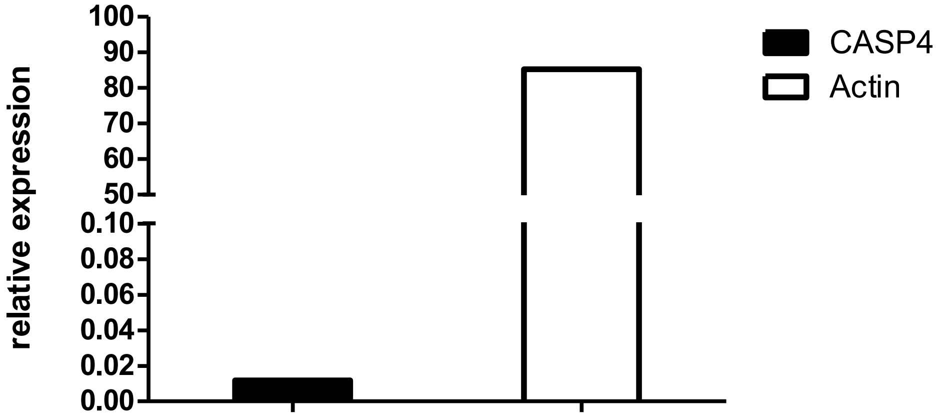

Supplementary Fig. S1. RT-PCR detect the mRNA level of caspase-4 in HEK293T cells.

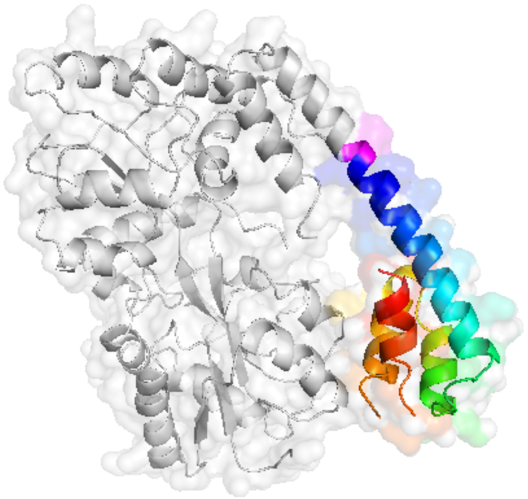

Supplementary Fig. S2. Cartoon diagram of caspase11-CARD with the MBP fusion protein in structure. Related to Figure 1. Cartoon diagram of caspase-11 CARD was shown with N-terminal blue to C-terminal red for chain C and MBP fusion protein in gray. The linker between CARD and MBP fusion protein was shown in rose red

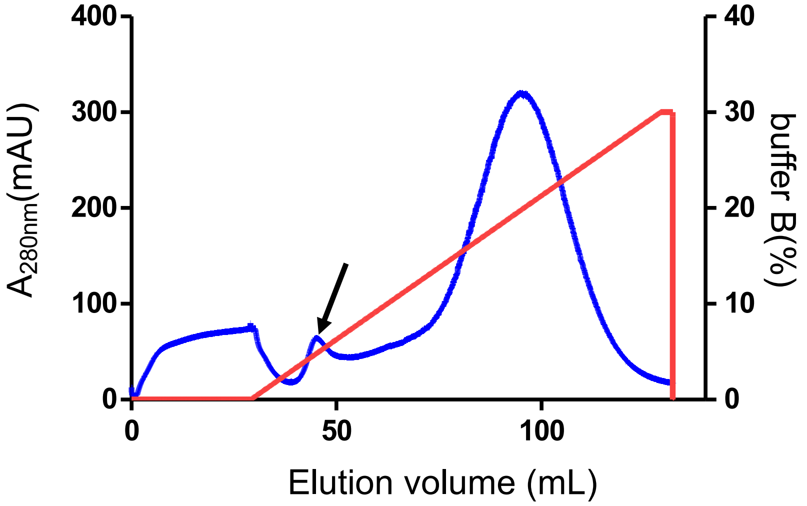

Supplementary Fig. S3. 2nd Nickel purification analysis of VLLV-CARD in liquid without MBP fusion protein

TEV enzyme was used to remove MBP fusion protein on N-terminal side of VLLV-CARD$^{aa11\text{-}101}$ protein. The arrow showed the VLLV-CARD$^{aa11\text{-}101}$ protein while 2nd Nickel purification.
